# P-2084. Construction of dominant peptide librarys of Mycobacterium tuberculosis-specific antigens and validation of their immunogenicity

**DOI:** 10.1093/ofid/ofae631.2240

**Published:** 2025-01-29

**Authors:** Yuanchun Li, Qiping Ge, Mengqiu Gao, Xiaoqing Liu, Lifan Zhang

**Affiliations:** Peking Union Medical College Hospital, Beijing, Beijing, China; Beijing Chest Hospital, Beijing, Beijing, China; Beijing Chest Hospital,, Beijing, Beijing, China; Peking Union Medical College Hospital, Chinese Academy of Medical Sciences and Peking Union Medical College, Beijing, Beijing, China; Peking Union Medical College Hospital,, Beijing, Beijing, China

## Abstract

**Background:**

Current immunological methods cannot accurately distinguish active tuberculosis (ATB) and latent tuberculosis infection (LTBI). The combination of latency-associated antigens with virulence antigens of Mtb enhances the discriminatory diagnostic ability. This study aims to screen and integrate dominant peptide segments of virulence factors and latency-associated antigens, verify their immunogenicity.

**Methods:**

ATB patients and LTBI healthy healthcare workers were enrolled, and PBMCs were isolated. Peptide pools of ESAT-6, CFP-10, Rv1733c and Rv2028c were used as stimuli. The frequencies of total IFN-γ secretion, total IL-2 secretion, and dual secretion of IFN-γ and IL-2 were measured using the FluoroSpot assay. Dominant peptide segments for library construction were screened based on the cytokine secretion characteristics of ATB and LTBI. Clinical T-SPOT.TB-positive patients were included for the verification of the immunogenicity of the newly combined peptide library.

**Results:**

Significant differences were found in the frequencies of specific T cells secreting IFN-γ, secreting IL-2, and dual-secreting IFN-γ and IL-2 between the ATB group and the LTBI group (p< 0.05) after stimulation with peptide pools from ESAT-6 and CFP-10, integrating six dominant peptide pools into the virulence antigen dominant peptide library. Similarly, six peptide pools from Rv1733c and Rv2028c were integrated into another dominant peptide library. The newly constructed library showed no significant differences (p >0.05) in the frequencies of total IFN-γ secretion, total IL-2 secretion, and dual secretion of IFN-γ and IL-2 compared to ESAT-6 combined with CFP-10, and the correlation coefficients were 0.947, 0.763, and 0.993, respectively. Similarly, the newly constructed latency-associated antigen library also showed no significant differences (p >0.05) in the frequencies of total IFN-γ secretion, total IL-2 secretion, and dual secretion of IFN-γ and IL-2 compared to Rv1733c combined with Rv2028c, and the correlation coefficients were 0.674, 0.562, and 0.692, respectively.

**Conclusion:**

The newly constructed librarys retain good immunogenicity and can be further used clinically to evaluate the accuracy of ATB and LTBI differential diagnosis.

**Disclosures:**

All Authors: No reported disclosures

